# Toxic Effects of Exposure to Phthalates on Cardiac Injury Biomarkers: Evidence from NHANES 1999–2004

**DOI:** 10.3390/metabo15020114

**Published:** 2025-02-10

**Authors:** He Li, Jifan Bu, Weilong Xing

**Affiliations:** 1 School of Civil Engineering, Southeast University, Nanjing 210096, China; ericlihe@seu.edu.cn (H.L.); 220224670@seu.edu.cn (J.B.); 2 Nanjing Institute of Environmental Science, Ministry of Ecology and Environment of China, Nanjing 210042, China

**Keywords:** phthalates, troponin, cardiac injury, NHANES, BKMR

## Abstract

**Background**: Humans are consistently and increasingly exposed to phthalate products, but the effect of the combined exposure to phthalates on myocardial injury remains largely unexplored. The present study aimed to explore the effect of the combined exposure to phthalates on myocardial injury. **Methods**: A total of 1237 male adults (aged ≥20) without coronary artery disease (CAD) from the National Health and Nutrition Examination Survey (NHANES) in 1999–2004 were included in the current study. Multiple linear regression, Bayesian kernel machine regression (BKMR), and a weighted quantile sum (WQS) model were employed to examine the associations of urinary phthalate metabolites with two cardiac injury biomarkers, including troponin T (TNT) and troponin I, using four highly sensitive assays (Abbott, Chicago, IL, USA; Siemens, Erlangen, Germany; and Ortho, Raritan, NJ, USA) (TNIA, TNIS, TNIO). **Results**: According to the linear regression analysis, mono-(3-carboxypropyl) phthalate (MCPP, a metabolite of di-n-octyl phthalate) was found to be positively associated with serum TNT; a positive association was found between mono-isobutyl phthalate (MiBP, a metabolite of di-isobutyl phthalate) and TNIA, as well as MiBP and TNIS. Mono-benzyl phthalate (MBzP, a metabolite of butyl benzyl phthalate) and MCPP were positively associated with serum TNIO. The BKMR analyses showed a positive overall relationship of serum TNT, TNIA, TNIS, and TNIO with increased concentrations of phthalate metabolites. The WQS model showed MCPP and MBzP were the top two contributors to being an increased risk for elevated TNT levels. MCPP and mono-ethyl phthalate (MEP, a metabolite of diethyl phthalate) were identified as the leading contributors to increased TNIA and TNIS. MCPP and MBzP were the dominant contributors to elevated TNIO. **Conclusions**: As a combined mixture, phthalate metabolites were positively associated with serum TNT and TNI among adults without CAD, indicating the potential toxic effect of phthalate exposure on cardiac injury.

## 1. Introduction

Phthalates, a class of synthetic chemical compounds, are widely utilized in a broad range of consumer and industrial products, including medical devices, cosmetics, plasticizers, lubricants, binders, and solvents [1]. Due to their physicochemical properties, phthalates are not covalently bound to the materials they are incorporated into, which allows them to easily leach out and migrate into the surrounding environment [2]. Consequently, humans are ubiquitously exposed to phthalates through multiple routes, such as oral ingestion, inhalation, dermal absorption, and, in some cases, parenteral exposure [3]. Cross-sectional studies have consistently demonstrated the pervasive nature of phthalate exposure, with the majority of phthalate metabolites being detectable in over 90% of urine samples examined among the general population in the United States [4]. This widespread exposure has raised significant concerns regarding the potential health risks associated with phthalates, particularly given their classification as endocrine-disrupting chemicals.

Phthalates have been inextricably linked to a range of adverse health outcomes, including teratogenicity, tumorigenesis, endocrine disruption, reproductive toxicity, and neurotoxicity [5,6,7,8,9]. Recent research had increasingly turned its attention to the cardiovascular toxicity associated with phthalate exposure. For example, experimental studies have shown that dibutyl phthalate (DBP) and butyl benzyl phthalate (BBP) could induce cardiac toxicity and disrupt the expression of heart-development-related genes in zebrafish embryos [10,11]. Similarly, di-(2-ethylhexyl) phthalate (DEHP), one of the most widely used phthalates, has been implicated in causing structural abnormalities in the fetal hearts of mice [12]. These findings align with a growing body of evidence suggesting that exposure to environmental chemicals, including phthalates, may elevate the risk of cardiovascular diseases (CVDs), such as coronary artery disease (CAD) and heart failure [13,14,15]. Despite these experimental findings, the epidemiological evidence linking phthalate exposure to cardiac injury or dysfunction in humans remains limited and poorly understood.

A key biomarker for assessing cardiac injury is troponin, a protein complex integral to muscle contraction in the heart that is widely used in clinical settings to diagnose myocardial injury. High-sensitivity troponin (hs-troponin), in particular, has emerged as a reliable indicator of subclinical cardiac injury and is increasingly being recognized as a predictor of mortality risk in the general population, as evidenced by data from the National Health and Nutrition Examination Survey (NHANES) [16]. The NHANES is a nationally cross-sectional survey conducted by the National Center for Health Statistics (NCHS) to assess the nutritional and health status of the American population. The NHANES public database integrates interviews, physical examinations, and laboratory analyses, offering comprehensive, multidimensional data on environmental exposure, diet, nutrition, and chronic diseases. Renowned for its high-quality data, large sample size, open accessibility, and long-term continuity, the NHANES serves as a vital resource for exploring the health effects of environmental exposures and provides invaluable insights to guide health promotion strategies [17,18]. Given the potential link between phthalate exposure and cardiovascular outcomes, further investigation is warranted to elucidate the relationship between phthalate exposure and hs-troponin levels in humans.

This study aimed to explore the association between phthalate exposure and hs-troponin concentrations in adults without pre-existing coronary artery disease (CAD) using data from the NHANES in 1999–2004. Notably, most prior research has focused on the effects of individual phthalates, neglecting the fact that phthalates often exist as mixtures in the environment and in human exposure scenarios. To address this gap, the present study employed advanced statistical approaches, including multivariate linear regression, Bayesian kernel machine regression (BKMR), and weighted quantile sum (WQS) regression models, to assess both the independent and combined effects of multiple phthalates on markers of cardiac injury. By leveraging these methodologies, this study sought to provide novel insights into the potential cardiovascular risks posed by phthalate exposure and to enhance our understanding of the broader health implications of environmental chemical exposure.

## 2. Materials and Methods

### 2.1. Data Sources and Study Population

**Figure 1:**
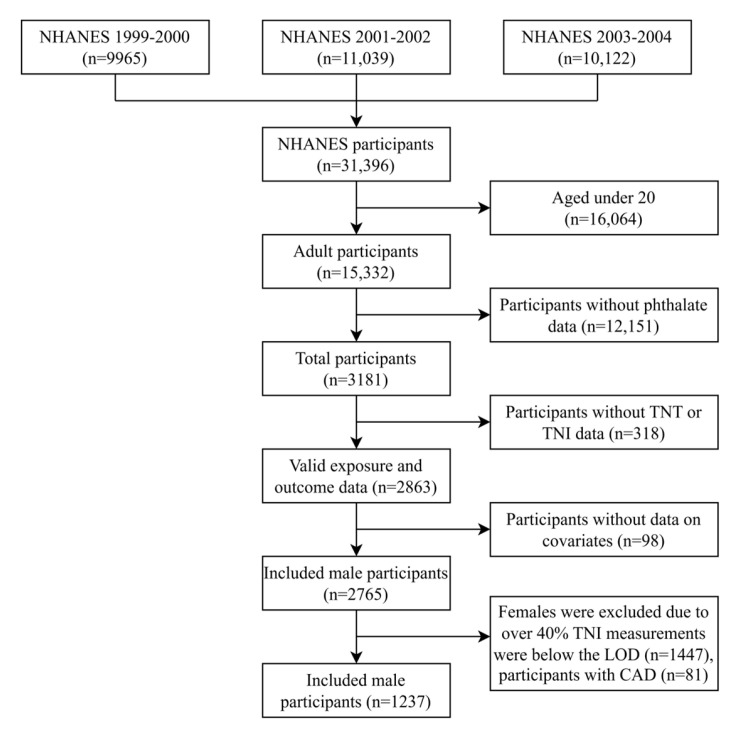
Flowchart of the population included in our final analysis from the NHANES 1999–2004. Abbreviations: NHANES: National Health and Nutrition Examination Survey; TNT: troponin T; TNI: troponin I; LOD: limit of detection; CAD: coronary artery disease.

The present analysis included 8 phthalate metabolite measurements and 4 hs-troponin biomarkers from 3 cycles (2009–2014) of the NHANES. The participant selection procedure is shown in Figure 1. Of 39316 participants, 15332 subjects aged at least 20 remained for subsequent research. A total of 2863 individuals with valid data for phthalates, urine creatinine, and troponin were included; 98 participants without data on covariates including age, race, BMI, education level, smoking, drinking, marital status, hypertension, diabetes, or hyperlipidemia were excluded. Over 40% of the serum troponin levels were below the LOD in female participants. Therefore, only 1318 male participants without CAD were included in the final research sample.

### 2.2. Urinary Phthalate Metabolite Analysis

Urine specimens were immediately stored at −20 °C until analysis. The processing and measurement methods were described in previous research [4]. Human urine samples were processed using enzymatic deconjugation of the glucuronides followed by solid-phase extraction. The eluate was concentrated, and the phthalate metabolites were chromatographically resolved by reversed-phase HPLC, detected by APCI tandem mass spectrometry (APCI-MS/MS), and quantified by isotope dilution. Assay precision was improved by incorporating 13C4-labeled internal standards for each of the seven analytes, as well as a conjugated internal standard to monitor deconjugation efficiency. A total of 12 kinds of phthalate metabolites in urine samples were measured, and their corresponding parent phthalate compounds are listed below:
Parent phthalate compoundsPhthalate metabolitesDi(2-ethylhexyl) phthalate (DEHP)Mono-(2-ethylhexyl) phthalate (MEHP)
Mono-(2-ethyl-5-hydroxyhexyl) phthalate (MEHHP)
Mono-(2-ethyl-5-oxohexyl) phthalate (MEOHP)Dibutyl phthalate (DBP)Mono-n-butyl phthalate (MBP)Diethyl phthalate (DEP)Mono-ethyl phthalate (MEP)Benzyl butyl phthalate (BBzP)Mono-benzyl phthalate (MBzP)Di-n-octyl phthalate (DNOP)Mono-(3-carboxypropyl) phthalate (MCPP)Di-isobutyl phthalate (DiBP)Mono-isobutyl phthalate (MiBP)Other phthalatesMono-n-octyl phthalate (MnOP)(less commonly studied phthalates)Mono-isononyl phthalate (MiNP)
Mono-cyclohexyl phthalate (MCHP)
Mono-n-methyl phthalate (MnMP)

Of the 12 phthalate metabolites, MCHP, MiNP, MnOP and MnMP were excluded from further study because over 40% of the measurements were below the limits of detection (LODs). The LODs of the remaining 8 metabolites ranged from 0.072 to 0.90 ng/mL (Table S1). Values below the LODs were replaced by the LOD divided by the square root of 2 (LOD/√2) in the present analysis. The molar concentrations of MEOHP, MEHP, and MEHHP were summarized to estimate the di (2-ethylhexyl) phthalate (DEHP) exposure level [19]. MECPP was not included because MECPP was not detected in the NHANES in 1999–2004.

### 2.3. Hs-Troponin Measurement

The samples were measured during 2018–2020 at the University of Maryland School of Medicine. hs-troponin T (TNT), hs-troponin I (Abbott, Chicago, IL, USA) (TNIA), Hs-troponin I (Siemens, Erlangen, Germany) (TNIS), and hs-troponin I (Ortho, Raritan, NJ, USA) (TNIO) were measured using a Roche Cobas e601, an Abbott ARCHITECT i2000SR, a Siemens Centaur XP and an Ortho Vitros 3600, respectively. The limit of detection (LOD) for TNT, TNIA, TNIS, and TNIO were 3, 1.7, 1.6, and 0.39 ng/L, respectively [16].

### 2.4. Covariates

Based on previous studies, all covariates that could influence the relationship of phthalate exposure with cardiac injury, including age (continuous), BMI (<25, 25–30, ≥30), race, education level, the ratio of family income to poverty (<1, ≥1), physical activity (no, moderate, vigorous), smoking status (current smoker, former smoker, and non-smoker) and alcohol consumption (drinker and non-drinker) were included in the models and regarded as potential confounders [20,21]. Hypertension was defined using blood pressure ≥140/90 mmHg or anti-hypertensive medication use. Hyperlipidemia and diabetes were defined as a self-reported diagnosis or current use of lipid-lowering or antidiabetic drugs. Participants with an estimated glomerular filtration rate (eGFR) of less than 60 mL/min/1.73 m^2^ were regarded as having chronic kidney disease.

### 2.5. Statistical Analysis

Urine creatinine (Cr) was used to calibrate phthalate metabolites levels. Then, all Cr-adjusted phthalate metabolites and cardiac injury indicators were natural logarithm (ln)-transformed to make these variables normally distributed. Spearman correlation analysis was performed to evaluate the correlations among these phthalate metabolite concentrations.

First, survey-weighed multivariate linear regression analysis adjusted for covariates was performed to explore the relationship between ln-transformed urinary phthalate metabolite concentrations and ln-transformed serum hs-troponin.

Second, a Bayesian kernel machine regression (BKMR) model was used to explore the individual and combined effects of phthalate metabolites on cardiac injury biomarkers and the potential interaction effect among these mixtures [22]. The posterior inclusion probabilities (PIPs) for each phthalate metabolite were calculated using the BKMR model and used to evaluate the significance of each metabolite, with a threshold of 0.5 in the current study. The BKMR models for cardiac injury indicators were based on the model below [23]:

$$Y_{i}=h\left(\sum {DEHP}_{i},{MBP}_{i},{MEP}_{i},{MBzP}_{i},{MCPP}_{i},{MiBP}_{i},\right)+\beta z_{i}+e_{i}$$

where $Y_{i}$ represents continuous serum TNT, TNIA, TNIS, and TNIO; *h*() represents the exposure–response function among these 6 phthalate metabolites; *β* is a coefficient; $z_{i}$ is the vector of covariates.

The weighted quantile sum (WQS) regression model is a quartile sum approach that we used to assess the effect of the combined exposure to 6 phthalate metabolites. All measured metabolites were taken into consideration in the WQS model. The WQS index was calculated and represents the mixed phthalates’ effect on increased serum troponin with a one-quantile increase in combined phthalates. The weight of each chemical was estimated by bootstrapping 10,000 sets and represents the contribution of each metabolite to the WQS index [24]. The WQS model was as follows:

$$g\left(\mu \right)=\beta_{0}+\beta_{1}\left(\sum_{i=0}^{c}\omega_{i}\varphi_{i}\right)+z^{\prime }\Phi {|}_{b}$$

$$WQS=\sum_{i=0}^{c}{\bar{\omega }}_{i}\varphi_{i}$$

where *β*_0_, *z′*, and Φ represent the intercept, matrix, and coefficient of the covariates; *c* represents the 6 chemicals in the analysis. $\omega_{i}$ represents the weight index of each chemical; $\varphi_{i}$ indicates the quartiles of the chemical score [24]. All models were adjusted for age, education, marital status, BMI, race, smoking, drinking, hypertension, hyperlipidemia, diabetes. *p* < 0.05 was regarded statistically significant. All analyses, including WQS and BKMR, were performed using R 4.2.2 software.

## 3. Results

### 3.1. Study Participants’ Characteristics

The characteristics of the 1237 male participants are summarized in Table 1. The mean age was 48.6 ± 18.0 years; 38.6% and 31.5% of them were overweight and obese. The majority of the participants were Mexican American (54.3%), well educated (46.0%), above the poverty level (78.4%), non-smokers (72.0%), drinkers (60.3%), and physically inactive (52.3%). A small proportion of participants had a history of hypertension (26.0%), hyperlipidemia (23.9%), diabetes (9.9%), or CKD (6.2%).

### 3.2. Distribution of Urinary Phthalate Metabolites and Serum Hs-Troponin

**Figure 2:**
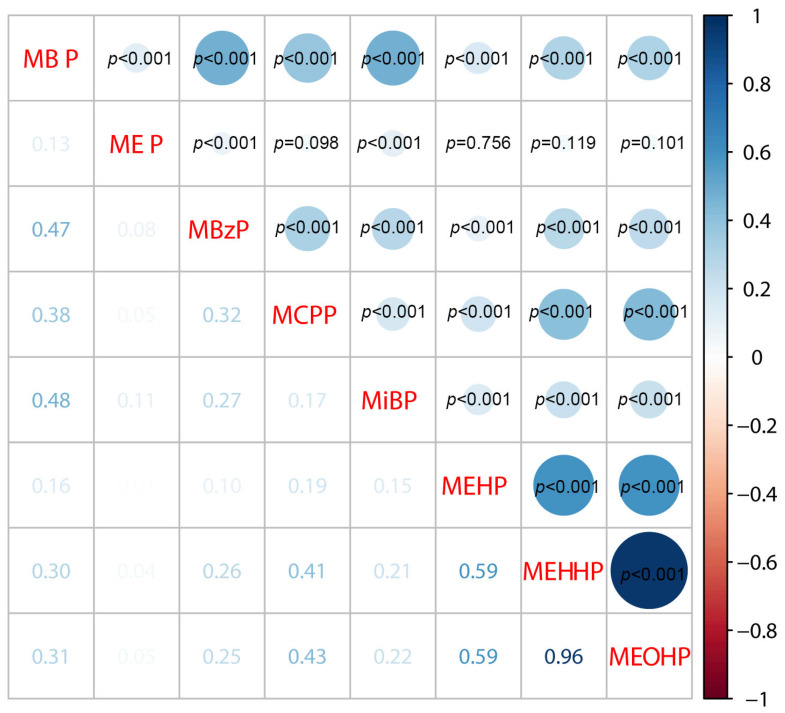
Spearman correlation analysis of urinary phthalate metabolite concentrations (μg/mmol Cr) in male adults (*n* = 1237), NHANES 1999–2016. Abbreviations: NHANES: National Health and Nutrition Examination Survey; MEHP: mono-(2-ethylhexyl) phthalate; MEHHP: mono-(2-ethyl-5-hydroxyhexyl) phthalate; MEOHP: mono-(2-ethyl-5-oxohexyl) phthalate; MBP: mono-n-butyl phthalate; MEP: mono-ethyl phthalate; MBzP: mono-benzyl phthalate; MCPP: mono-(3-carboxypropyl) phthalate; MiBP: mono-isobutyl phthalate. Spearman correlation analysis was performed using R 4.2.2.

The detection rates of the majority of phthalate metabolites were >98%, while the detection rate of MEHP was 83.91%. The distribution of the urinary phthalate metabolites and serum hs-troponin are presented in Table 2. The median concentrations of MEHP, MEHHP, MEOHP, ∑DEHP, MBP, MEP, MBzP, MCPP, and MiBP were 0.26, 1.59, 1.02, 2.88, 1.52, 12.05, 0.83, 0.22, and 0.28 μg/mmol Cr. The median levels of hs-TNT, hs-TNIA, hs-TNIO, and hs-TNIS were 6.61, 2.20, 3.36, and 0.69 ng/L. Spearman’s rho was employed to examine the correlations among the eight phthalate metabolites in this study, with the results shown in Figure 2. The strongest correlation was found between MEHHP and MEOHP (r = 0.96, *p* < 0.001), followed by MEHP and MEOHP (r = 0.59, *p* < 0.001), as well as MEHHP and MEHP (r = 0.59, *p* < 0.001). These strong correlations likely stemmed from the fact that MEHHP, MEOHP, and MEHP are all metabolites of DEHP. Additionally, a notable correlation was observed between MiBP and MBP (r = 0.48, *p* < 0.001), potentially due to their structural similarity as isomers as well as the isomeric nature of their parent compounds. Furthermore, MEOHP and MEHHP exhibited moderate correlations with MCPP (r = 0.43 and r = 0.41, respectively, *p* < 0.001), possibly explained by the structural similarity between the parent compound of MEOHP and MEHHP (DEHP) and that of MCPP (DNOP), as both are isomers. Therefore, the BKMR model was constructed for subsequent analysis to avoid the interference of multicollinearity.

### 3.3. Linear Regression Analysis of Associations Between Phthalate Metabolites and Hs-Troponin

**Figure 3:**
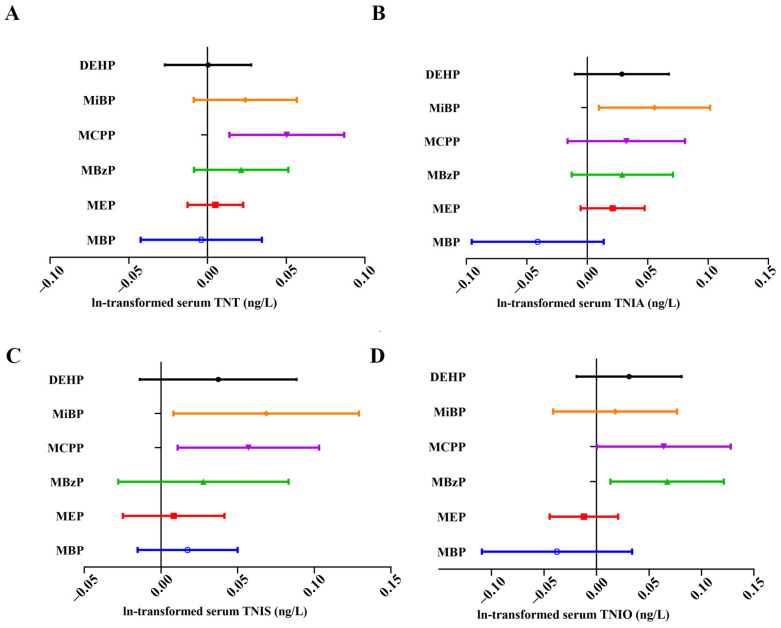
Association between individual urinary phthalate metabolites with serum TNT (**A**), TNIA (**B**), TNIS (**C**), and TNIO (**D**) levels based on survey-weighted linear regression. Black, orange, purple, green, red, and blue represent DEHP, MiBP, MCPP, MBzP, MEP, and MBP, respectively. Abbreviations: DEHP: di(2-ethylhexyl) phthalate; MBP: mono-n-butyl phthalate; MEP: mono-ethyl phthalate; MBzP: mono-benzyl phthalate; MCPP: mono-(3-carboxypropyl) phthalate; MiBP: mono-isobutyl phthalate; TNT: troponin T; TNIA: troponin I (Abbott); TNIS: troponin I (Siemens); TNIO: troponin I (Ortho). Linear regression analysis was performed and forest plot was created using R 4.2.2.

The result of the survey-weighed linear regression analysis is shown in Figure 3 and Table S2. A 1-unit increase in ln-MCPP was associated with a 0.064 (95% CI: 0.009–0.078, *p* = 0.013)-unit increase in ln-TNT. A 1-unit increase in ln-MiBP was associated with a 0.064 (95% CI: 0.006–0.097, *p* = 0.028)-unit increase in ln-TNIA and 1 0.069 (95% CI: 0.008–0.129, *p* = 0.027)-unit increase in ln-TNIS. A 1-unit increase in ln-transformed MBzP and MCPP levels was associated with 0.068 (95% CI: 0.013–0.121, *p* = 0.015)- and 0.057 (95% CI: 0.004–0.131, *p* = 0.037)-unit increases in ln-TNIO. Consistently, previous studies have found that these three metabolites are closely associated with rheumatoid arthritis, IL-6, C-reactive protein, and other inflammatory markers [25,26]. We speculate that these metabolites may induce myocardial injury by modulating inflammatory responses. However, the results of univariate analysis are prone to the influence of various confounding factors, and the interactions among different metabolites may further complicate the findings. Therefore, we subsequently utilized the BKMR model to investigate the overall mixed effects of phthalates on myocardial injury.

### 3.4. BKMR Analysis of Associations Between Phthalate Mixtures and Serum Hs-Troponin

**Figure 4:**
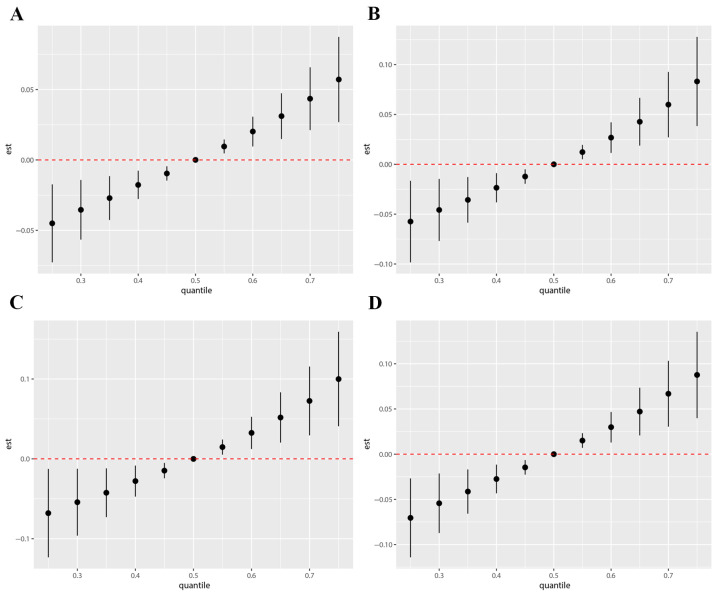
The BKMR models of exposure to mixed phthalates on cardiac injury. The BKMR models of the overall effects of exposure to mixtures of phthalate metabolites at particular percentiles (5th to 95th) on serum TNT (**A**), TNIA (**B**), TNIS (**C**), and TNIO (**D**) concentrations as compared with their medians (P50). The red dotted line represents the null effect (est = 0), serving as a reference line to indicate no association. Abbreviations: BKMR: Bayesian kernel machine regression; TNT: troponin T; TNIA: troponin I (Abbott); TNIS: troponin I (Siemens); TNIO: troponin I (Ortho). The BKMR model was applied using R 4.2.2.

The BKMR model showed significant dose-– relationships between mixed phthalate metabolites levels and hs-troponin. In comparison with the median level, TNT, TNIA, TNIS, and TNIO increased remarkably when the combined phthalate metabolites were at or above the 55th percentile (Figure 4). The PIP for each metabolite was calculated to evaluate their contribution to the mixtures’ effect on cardiac injury. MCPP was identified as the dominant contributor to the higher TNT concentrations in the BKMR analysis (PIP = 0.9704). MiBP had the highest PIP with regard to increased TNIA (PIP = 0.4780) and TNIS (PIP = 0.4512) compared with the others. MBzP (PIP = 0.6556) and MCPP (PIP = 0.5168) made the largest contribution to the positive association with TNIO. The PIP of each metabolite in the effect of the combined exposure to phthalates on TNT, TNIA, TNIS, and TNIO is listed in Table S3.

**Figure 5:**
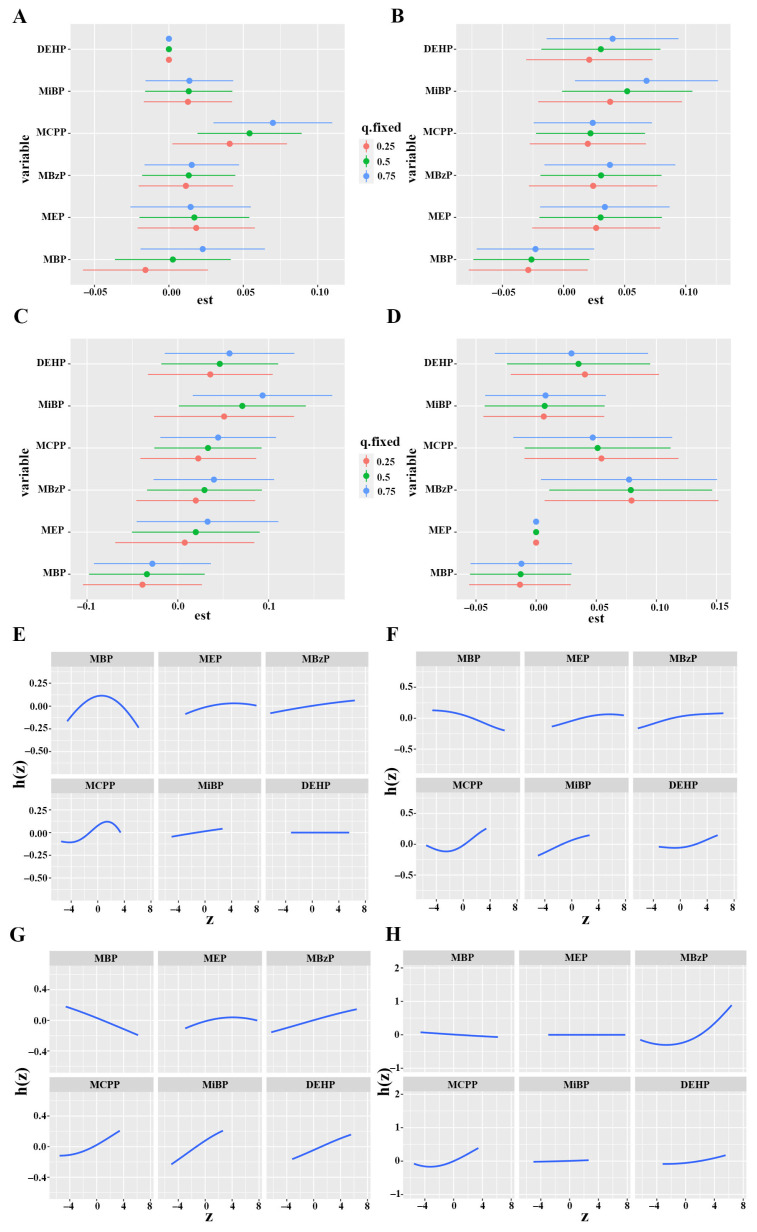
Association of individual phthalate metabolites with serum TNT (**A**), TNIA (**B**), TNIS (**C**), and TNIO (**D**) while other phthalate metabolites were fixed at the 25th, 50th, and 75th percentiles. Univariate exposure–response function of individual urinary phthalate metabolite concentrations with serum TNT (**E**), TNIA (**F**), TNIS (**G**), and TNIO (**H**) per BKMR models while fixing the other phthalate metabolites at the 25th, 50th, and 75th percentiles. Models were adjusted by age, BMI, race, education, marital status, smoking, alcohol consumption, hypertension, hyperlipidemia, diabetes. Abbreviations: DEHP: di(2-ethylhexyl) phthalate; MBP: mono-n-butyl phthalate; MEP: mono-ethyl phthalate; MBzP: mono-benzyl phthalate; MCPP: mono-(3-carboxypropyl) phthalate; MiBP: mono-isobutyl phthalate; TNT: troponin T; TNIA: troponin I (Abbott); TNIS: troponin I (Siemens); TNIO: troponin I (Ortho); BKMR: Bayesian kernel machine regression; BMI: body mass index. Dose–response relationship curve was created using R 4.2.2.

The univariate effect of each phthalate metabolite on cardiac injury was explored when the others were fixed at certain quartiles. MCPP and MBzP were identified as the sole metabolites exhibiting a significantly positive effect on serum TNT and TNIO, respectively. The positive effects of MiBP on serum TNIA were statistically significant when they reached their 75th percentile. The effect of MiBP on serum TNIS augmented when the others increased from the 25th to the 50th percentile, and MiBP was found to be significantly positively associated with TNIS concentrations when the others were fixed at the median and 75th percentiles. MBzP was positively correlated with serum TNIO concentrations at the 25, 50, and 75th percentiles (Figure 5A–D).

Figure 5 presents the univariate exposure–response functions when the others were fixed at their median levels. MBP and MCPP showed an increasing and then declining relationship with serum TNT, MEP, MBzP, and MiBP, which were positively associated with serum TNT. Only MBP was found to be negatively corelated with TNIA and TNIS; the other five metabolites were significantly associated with increased serum TNIA and TNIS. Additionally, significantly positive associations were found between MBzP and TNIO, as well as MCPP and TNIO (Figure 5E–H).

**Figure 6:**
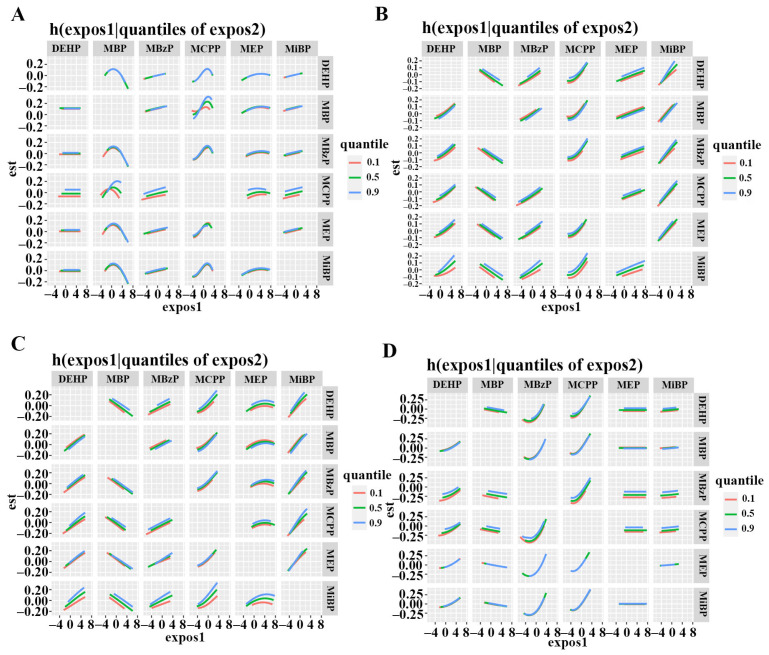
Bivariate exposure–response functions for serum TNT (**A**), TNIA (**B**), TNIS (**C**) and TNIO (**D**) for each phthalate metabolites were assessed with BKMR models when exposure to metabolites was at different quantiles (10th, 50th, and 90th), and other metabolites were fixed at their median levels. BKMR models were adjusted for age, BMI, race, education, marital status, smoking, alcohol consumption, hypertension, hyperlipidemia, diabetes. Abbreviations: DEHP: di(2-ethylhexyl) phthalate; MBP: mono-n-butyl phthalate; MEP: mono-ethyl phthalate; MBzP: mono-benzyl phthalate; MCPP: mono-(3-carboxypropyl) phthalate; MiBP: mono-isobutyl phthalate; TNT: troponin T; TNIA: troponin I (Abbott); TNIS: troponin I (Siemens); TNIO: troponin I (Ortho); BKMR: Bayesian kernel machine regression; BMI: body mass index. Dose–Response relationship curves were created using R 4.2.2.

For TNT, the results of BKMR indicated there might be interactions for TNT between MBP and MCPP. The positive slope of MiBP tended to be steep with the increasing percentile concentration of MBP, indicating a potential positive interaction between MiBP and DEHP. Additionally, the positive slope of DEHP, MBzP, MCPP, and MEP for TNIS tended to be sharp as the percentile concentrations of MiBP increased. Potential interactions for TNIO were found between DEHP and MBzP, MCPP and MBzP, as well as DEHP and MCPP (Figure 6).

### 3.5. WQS Analysis of Associations Between Phthalate Mixtures and Troponin

As displayed in Figure S1, MCPP (51.0%), MBzP (21.6%), and MEP (19.5%) were the top three contributors to elevated TNT after adjusting for confounding factors. MCPP (33.2%) and MEP (32.0%) had the highest weights for increased TNIA. Similarly, MCPP (42.1%) and MEP (25.5%) were the leading contributors to increased serum TNIS. In addition, MCPP (59.9%) and MBzP (25.3%) were the leading contributors to TNIO. The details of the WQS weights in the WQS models are listed in Figure S1. The scatterplot showing the association between the WQS index, TNT (A), TNIA(B), TNIS(C), and TNIO(D) through WQS model is presented in Figure S2.

## 4. Discussion

As far as we know, this is the first study to examine the effects of the relationship between the combined exposure to phthalates and cardiac injury in adults without CAD in a study population from the NHANES 1999–2004 using linear regression analysis, BKMR, and WQS models. In multivariate linear regression analysis, MCPP was found to be positively associated with serum TNT, MiBP was associated with increased TNIA and TNIO, and MBzP and MCPP correlated with an increase in TNIO. The BKMR models showed the exposure to various phthalates was significantly positively associated with serum TNT, TNIA, TNIS, and TNIO. From these research findings, MCPP was identified as the main contributor to the increased concentrations of serum troponin. MBzP and MEP were another two crucial phthalate metabolites leading to cardiac injury.

Troponin is released into the blood circulation upon cardiac injury and is regarded as a classic cardiac injury biomarker with high sensitivity and specificity [27]. Troponin T and I are ideal myocardial damage markers because they are specifically expressed in the heart [28]. As such, hs-troponin detection methods have been developed to detect minor cardiac damage. Intriguingly, the studies showing that environmental pollution, in addition to traditional risk factors, including hypertension, hyperlipidemia, diabetes, and smoking, could also increase the risk of cardiac injury are accumulating. For instance, traffic-related air pollution (TRAP) was relevant to increased biomarkers of cardiac injury and heart failure, including hs-troponin I and B-type natriuretic peptide [29]. Another cross-sectional study that included participants with myocardial infarction (MI) reported a significant association between TRAP exposure, higher MI risk, and cardiac troponin T levels [30]. Additionally, occupational exposure to fire emissions altered the expression of cardiac troponin T [31]. However, evidence of the role of phthalate exposure in cardiac injury is limited. Therefore, continuous troponin T and I were used in the current research to examine the effects of phthalate exposure on cardiac injury among participants without a known CAD history.

Phthalates are a class of endocrine-disrupting chemicals, the exposure to which has been shown to be correlated with adverse human health outcomes, such as reproductive toxicity [32] and dysregulated lipid metabolism [33]. Of note, compelling epidemiological and experimental evidence revealed that the exposure to phthalates contributes to a higher risk of cardiovascular diseases (CVDs). For example, DEHP exposure could induce cardiac birth defects [34] as well as initiate and deteriorate the development of CVDs through disturbing inflammation and oxidative stress [35]. DBP was identified as an environmental cardiotoxic pollutant that functions through the induction of mitochondrial damage and the activation of inflammation [36]. Di-isononyl phthalate (DINP) was associated with higher blood pressure and heart rate [37]. In a cross-sectional study that included 675 diabetic patients from China, urinary MEP and MiBP concentrations were found to be positively associated with 13.8% and 36.9% higher risks of CVDs [38]. Furthermore, exposure to MiBP was shown to increase the risk of CADs. Patients with coronary heart disease (CHD) had higher urinary MiBP concentrations compared with those without CHD [39]. Serum MiBP levels were associated with the echogenicity of carotid artery plaques, indicating the role of MiBP in atherosclerosis [40]. The current study firstly revealed a higher risk of cardiac injury upon phthalates’ exposure among the general population. However, the underlying mechanism through which phthalates cause cardiac injury remains unexplored.

The present study indicated that MCPP, MEP, and MBzP were the leading contributors to an increased risk for cardiac injury. Previous researches have revealed that the exposure to these metabolites might have an influence on human health. For instance, Ivy et al. [41] found that urinary MCPP and MBzP levels were associated with increased hearing loss risk in those aged over 50. Another epidemiological report noted that higher MCPP levels in urine samples produced positive effects on increased obesity in children [42]. Zhang and colleagues found that MEP was related to decreased heart rate variability [43]. The findings from a longitudinal panel study indicated that MEP was inversely associated with heart rate variability, an indicator of cardiac autonomic imbalance [43]. The present study found an association between these three phthalate metabolites and cardiac injury, but it could not determine causation.

Our study has several strengths. First of all, we, for the first time, explored the effect of phthalate exposure on serum hs-troponin concentrations among the general population without CAD. In addition, both the individual and mixed effects of these phthalate metabolites were evaluated using three different models. Meanwhile, the current research has several limitations. First of all, the cross-sectional nature of this study prevented us from determining the causal relationships between phthalate exposure and cardiac injury. A large-scale prospective study and experimental studies are warranted to further verify the causal relationship between them and the underlying mechanisms. Secondly, some phthalate metabolite variables such as MECPP were not available in the data from the NHANES 1999–2004. Furthermore, four phthalate metabolites were not included in the research due to >40% of the data being invalid. Thirdly, the current study only included male subjects because a considerable percentage of the serum troponin data were absent for female participants. Excluding female participants limits the applicability of our findings to the general population. Finally, factors such as dietary patterns, occupational exposure, and co-exposures to other environmental pollutants may influence both phthalate exposure and cardiac injury biomarkers. In the future, clinical studies with holistic data on phthalate exposure will be carried out to clarify its effect on cardiac injury.

Based on the findings of this study, we offer the following recommendations for policymakers, clinicians, and researchers: Policymakers should prioritize the development and promotion of phthalate alternatives in industrial and consumer applications, with a particular focus on reducing exposure in vulnerable populations, such as children and individuals with pre-existing cardiovascular conditions. Clinicians should recognize the potential cardiovascular risks associated with phthalate exposure and consider screening for environmental exposures in clinical practice. Future research should emphasize longitudinal and experimental studies to establish the causal relationships between phthalate exposure and cardiac injury.

## 5. Conclusions

In conclusion, phthalate exposure was significantly positively associated with cardiac injury. The effects of exposure to various phthalates on cardiac injury were enhanced by each other according to the interaction analysis of the BKMR model. There was a significant positive effect on increased serum troponin level when these phthalate metabolites were regarded as a whole. MCPP, MEP, and MBzP were the top three contributors to cardiac injury among these metabolites. Our findings could contribute to the development of evidence-based regulatory policies and public health interventions aimed at mitigating the adverse effects of phthalates on potential cardiac injury.

## Figures and Tables

**Table 1:** Baseline characteristics of the included male participants in NHANES 1999–2004 (*n* = 1237).

Characteristics	Overall
Age	48.6 ± 18.0
20 ≤ age < 40	431 (34.8)
40 ≤ age < 60	422 (34.1)
age ≥ 60	384 (31.1)
BMI, *n* (%)	
BMI < 25	370 (29.9)
25 ≤ BMI < 30	477 (38.6)
BMI ≥ 30	390 (31.5)
Race, *n* (%)	
Mexican American	655 (54.3)
Other Hispanic	236 (18.6)
Non-Hispanic White	264 (21.3)
Non-Hispanic Black	31 (2.5)
Other race—including multi-racial	51 (4.1)
Education level, *n* (%)	
Less than high school	369 (29.8)
High school	299 (24.2)
College or above	569 (46.0)
PIR, *n* (%)	
<1	267 (21.6)
≥1	970 (78.4)
Smoking status, *n* (%)	
Current smoker	142 (11.5)
Former smoker	204 (16.5)
Non-smoker	891 (72.0)
Drinking status, *n* (%)	
Yes	795 (60.3)
No	523 (39.7)
Physical activity, *n* (%)	
No	647 (52.3)
Moderate	249 (20.1)
Vigorous	341 (27.6)
Hypertension, *n* (%)	
Yes	322 (26.0)
No	915 (74.0)
Hyperlipidemia, *n* (%)	
Yes	296 (23.9)
No	941 (76.1)
Diabetes, *n* (%)	
Yes	122 (9.9)
No	1115 (90.1)
CKD, *n* (%)	
Yes	77 (6.2)
No	1160 (93.8)
Urine creatinine (mg/dL)	152.6 ± 88.1

Abbreviations: BMI: body mass index; PIR: poverty income ratio; CKD: chronic kidney disease.

**Table 2:** The distribution of urinary phthalate metabolites, troponin T, and troponin I, NHANES 1999–2004 (*n* = 1237).

Urine Metabolites (μg/mmol Cr)	>LOD (%)	Percentage		
		P25	P50	P75
MEHP	83.91	0.12	0.26	0.63
MEHHP	99.76	0.93	1.59	3.10
MEOHP	99.43	0.62	1.02	1.98
∑DEHP ^a^	/	1.68	2.88	5.46
MBP	99.35	0.99	1.52	2.58
MEP	100.00	4.55	12.05	35.57
MBzP	99.51	0.46	0.83	1.49
MCPP	99.27	0.15	0.22	0.35
MiBP	98.54	0.16	0.28	0.50
Troponin (ng/L)				
TNT	98.63	5.03	6.61	10.32
TNIA	68.31	1.50	2.20	3.60
TNIS	81.97	2.02	3.36	5.73
TNIO	67.74	0.27	0.69	1.49

^a^ ΣDEHP indicates the creatinine corrected molar sum of DEHP metabolites including MEHP, MEHHP, and MEOHP (expressed as MEHP, molecular weight 278). Abbreviations: DEHP: di(2-ethylhexyl) phthalate; MEHP: mono-(2-ethylhexyl) phthalate; MEHHP: mono-(2-ethyl-5-hydroxyhexyl) phthalate; MEOHP: mono-(2-ethyl-5-oxohexyl) phthalate; MBP: mono-n-butyl phthalate; MEP: mono-ethyl phthalate; MBzP: mono-benzyl phthalate; MCPP: mono-(3-carboxypropyl) phthalate; MiBP: mono-isobutyl phthalate; TNT: troponin T; TNIA: troponin I (Abbott); TNIS: troponin I (Siemens); TNIO: troponin I (Ortho).

## Data Availability

Publicly available datasets were analyzed in this study. These data can be found at https://www.cdc.gov/nchs/nhanes/?CDC_AAref_Val=https://www.cdc.gov/nchs/nhanes/index.htm (accessed on 19 April 2023).

## Supplementary Materials

The following supporting information can be downloaded at: https://www.mdpi.com/article/10.3390/metabo15020114/s1, Figure S1: Weighted quantile sum (WQS) weights in the WQS regression model between TNT (A), TNIA (B), TNIS (C), TNIO (D), and WQS index of each phthalate mixture; Figure S2: A scatterplot showing the association between the WQS index, TNT (A), TNIA(B), TNIS (C), and TNIO (D) per WQS model; Table S1: The limit of detection (LOD, in ng/mL) for each phthalate metabolite; Table S2: Association between log-transformed phthalate metabolites and cardiac injury indicators; Table S3: Posterior inclusion probabilities (PIPs) using Bayesian kernel machine regression (BKMR) model (n = 1237).

## Abbreviations

The following abbreviations are used in this manuscript:

CAD	Coronary artery disease
NHANES	National Health and Nutrition Examination Survey
BKMR	Bayesian kernel machine regression
WQS	Weighted quantile sum
TNT	Troponin T
MCPP	Mono-(3-carboxypropyl) phthalate
MiBP	Mono-isobutyl phthalate
MBzP	Mono-benzyl phthalate
DEHP	Di-(2-ethylhexyl) phthalate
DBP	Dibutyl phthalate
BBP	Butyl benzyl phthalate
MEHP	Mono-(2-ethylhexyl phthalate
MEHHP	Mono-(2-ethyl-5-hydroxyhexyl) phthalate
MEOHP	Mono-(2-ethyl-5-oxohexyl) phthalate
MBP	Mono-n-butyl phthalate
MEP	Mono-ethyl phthalate
MnOP	Mono-n-octyl phthalate
MiNP	Mono-isononyl phthalate
MCHP	Mono-cyclohexyl phthalate
MnMP	Mono-n-methyl phthalate
LOD	Limit of detection
eGFR	Estimated glomerular filtration rate
