# Supplementary material for: Toxic Effects of Exposure to Phthalates on Cardiac Injury Biomarkers: Evidence from NHANES 1999–2004
Source: Metabolites. 2025 Feb 10;15(2):114. doi: 10.3390/metabo15020114 (PMC11857284; doi:10.3390/metabo15020114)
Supplement: Supplementary file 1 [file metabolites-15-00114-s001.zip › metabolites-3454603-supplementary.pdf]

Table S1. The Limit of detection (LOD, in ng/mL) for each phthalate metabolite.

Phthalate metabolites	LOD (ng/mL)
MEHP	0.90
MEHHP	0.32
MEOHP	0.45
MBP	0.40
MEP	0.264
MBzP	0.072
MCPP	0.16
MiBP	0.26

Table S2. Association between log-transformed phthalate metabolites and cardiac injury indicators.

Phthalate metabolite	β	95% CI	p value
TNT			
∑DEHP	-0.005	-0.024-0.030	0.838
MBP	-0.005	-0.042-0.035	0.865
MEP	0.008	-0.014-0.021	0.706
MBzP	0.030	-0.012-0.046	0.253
**MCPP**	**0.064**	**0.009-0.078**	**0.013**
MiBP	0.032	-0.012-0.052	0.214
TNIA			
∑DEHP	0.035	-0.013-0.064	0.197
MBP	-0.046	-0.093-0.016	0.165
MEP	0.031	-0.009-0.041	0.213
MBzP	0.036	-0.016-0.067	0.225
MCPP	0.036	-0.018-0.079	0.215
**MiBP**	**0.064**	**0.006-0.097**	**0.028**
TNIS			
∑DEHP	0.040	-0.015-0.087	0.171
MBP	-0.044	-0.119-0.027	0.213
MEP	0.011	-0.026-0.040	0.684
MBzP	0.030	-0.029-0.082	0.342
MCPP	0.041	-0.021-0.109	0.183
**MiBP**	**0.069**	**0.008-0.129**	**0.027**
TNIO			
∑DEHP	0.031	-0.019-0.081	0.230
MBP	-0.034	-0.111-0.032	0.274
MEP	-0.015	-0.043-0.022	0.514
**MBzP**	**0.068**	**0.013-0.121**	**0.015**
**MCPP**	**0.057**	**0.004-0.131**	**0.037**
MiBP	0.017	-0.041-0.077	0.547

Table S3. Posterior inclusion probabilities (PIPs), using Bayesian kernel machine regression (BKMR) model (n=1237).

Analyte	TNT	TNIA	TNIS	TNIO
∑DEHP	0.0000	0.2332	0.2016	0.1172
MBP	0.3748	0.0704	0.0596	0.0104
MEP	0.0816	0.0432	0.1140	0.0000
MBzP	0.0212	0.1536	0.0864	0.6556
MCPP	0.9704	0.2656	0.2852	0.5168
MiBP	0.0108	0.4780	0.4512	0.0144

All models were adjusted by age, race, BMI, smoking, drinking, hypertension, hyperlipidemia, diabetes, education, marital status. Both phthalate metabolites and troponins were Ln transformed.

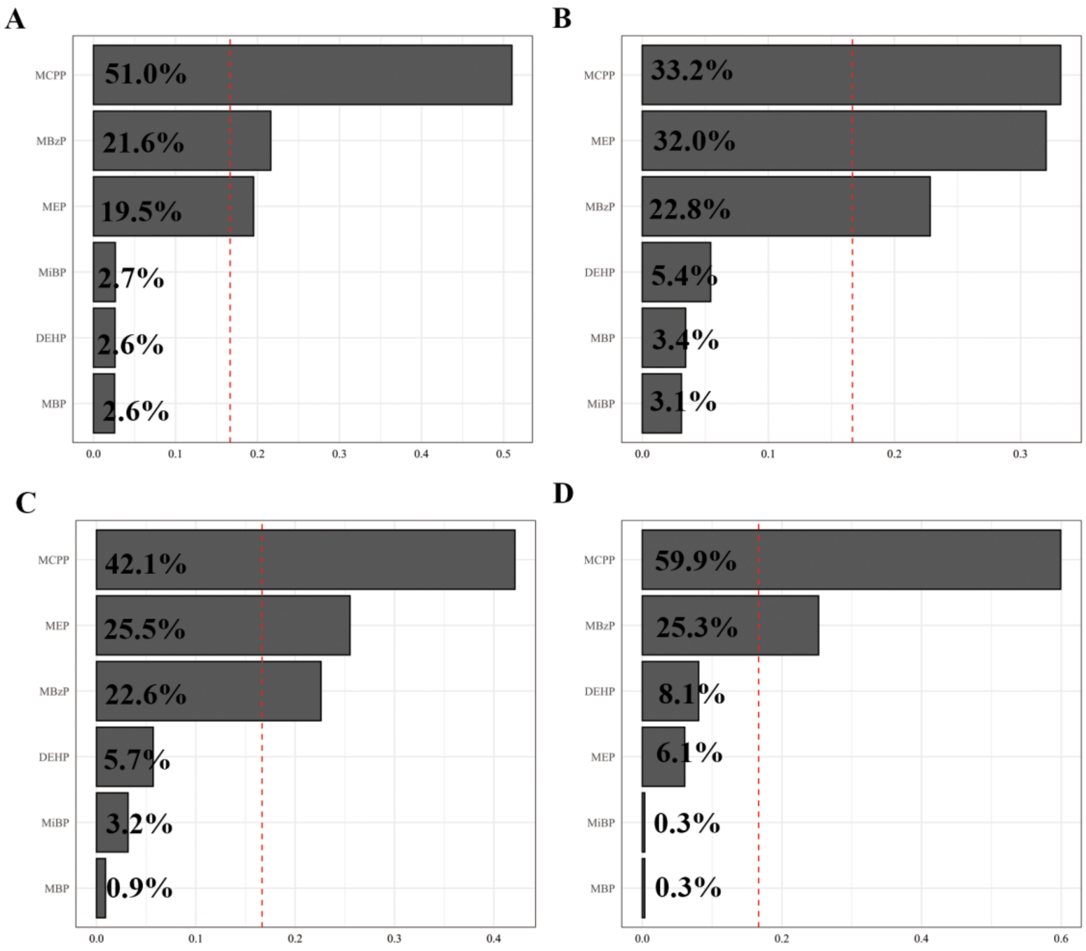

Figure S1. Weighted quantile sum (WQS) weights in the WQS regression model between TNT (A), TNIA (B), TNIS (C), TNIO (D) and WQS index of each phthalate mixtures.

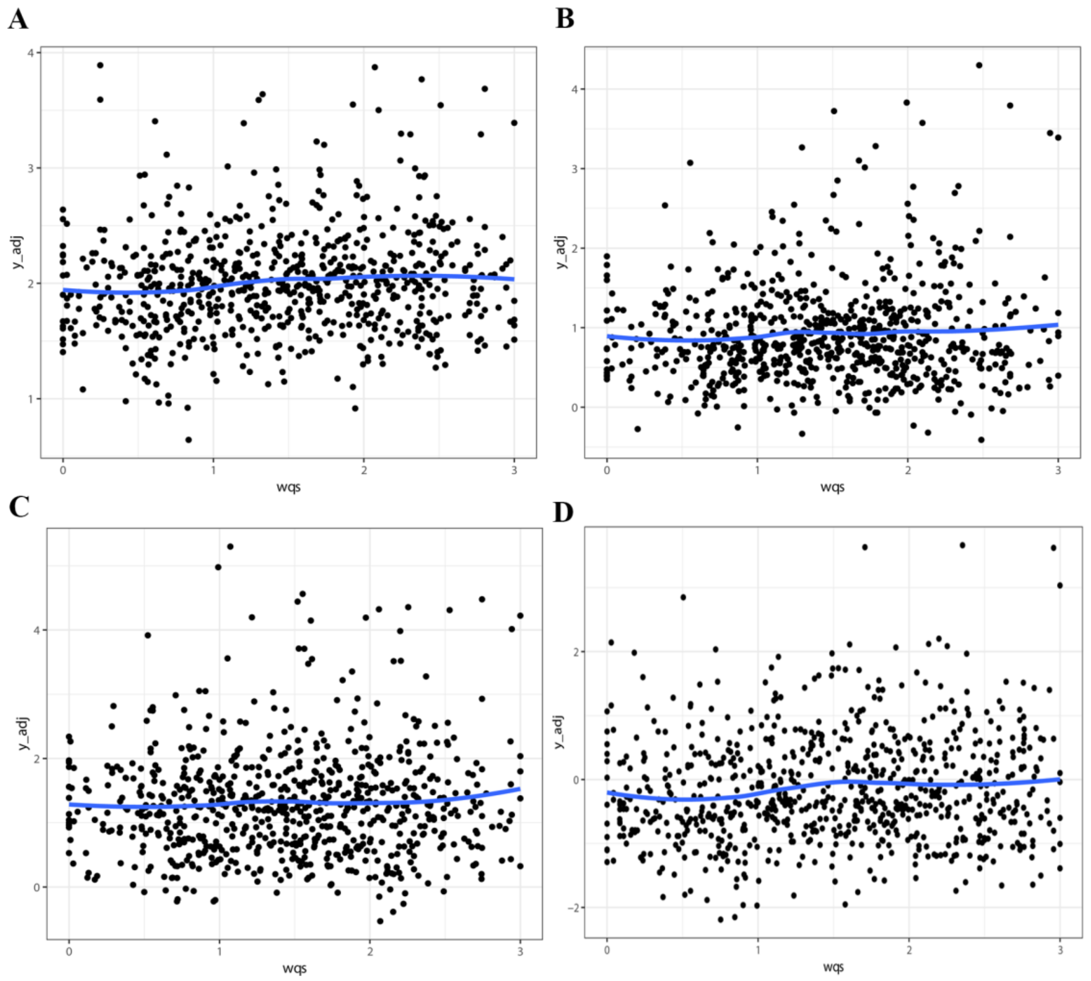

Figure S2. The scatterplot showing the association between WQS index, TNT (A), TNIA(B), TNIS(C), and TNIO(D) through WQS model.
